# Unfolding of ^239^Pu–Be and ^252^Cf neutron energy spectra using passive multi-layer neutron spectrometer

**DOI:** 10.1093/rpd/ncad169

**Published:** 2023-10-11

**Authors:** Maciej Maciak, Szymon Domański, Piotr Tulik, Katarzyna Tymińska, Michał A Gryziński

**Affiliations:** National Centre for Nuclear Research, Nuclear Facilities Operations Department, Radiological Metrology and Biomedical Physics Division, 7 Andrzeja Sołtana Street, 05-400 Otwock, Poland; Faculty of Mechatronics, Institute of Metrology and Biomedical Engineering, Warsaw University of Technology, 8 św. Andrzeja Boboli Street, 02-525 Warsaw, Poland; National Centre for Nuclear Research, Nuclear Facilities Operations Department, Radiological Metrology and Biomedical Physics Division, 7 Andrzeja Sołtana Street, 05-400 Otwock, Poland; National Centre for Nuclear Research, Nuclear Facilities Operations Department, Radiological Metrology and Biomedical Physics Division, 7 Andrzeja Sołtana Street, 05-400 Otwock, Poland; National Centre for Nuclear Research, Nuclear Facilities Operations Department, Radiological Metrology and Biomedical Physics Division, 7 Andrzeja Sołtana Street, 05-400 Otwock, Poland; National Centre for Nuclear Research, Nuclear Facilities Operations Department, Radiological Metrology and Biomedical Physics Division, 7 Andrzeja Sołtana Street, 05-400 Otwock, Poland

## Abstract

In the study, the passive multi-layer neutron spectrometer, based on thermoluminescence detectors, was tested in a calibration laboratory with ^239^Pu–Be and ^252^Cf isotopic sources. MCNP code was used for the calculation of the response functions for the neutron energy range from 1 meV to 100 MeV. It was also utilised for initial guess spectra calculations. Deconvolution was performed with MAXED and GRAVEL deconvolution codes resulting in the neutron spectra defined at the measuring point in the calibration laboratory.

## Introduction

Passive neutron spectrometers using activation foils or thermoluminescent detectors are used widely around the world in many different forms^(1)^. Usually, they consist of a single-moderator sphere/cylinder made of high-density polyethylene and a few layers containing small thermal neutron-sensitive detectors placed uniformly at different depths. Systems based on integral detectors: avoid dead-time losses, especially in pulsed fields; are insensitive to high electromagnetic noise; can extract the gamma component coming from high photon contamination of the field. These types of neutron spectrometry systems are generally based on the principle proposed by Bramblett *et al*.^(2)^, where a small ^6^LiI(Eu) scintillator placed at the center of polyethylene moderating spheres with different diameters was used. In such a way the thermal-neutron-sensitive detector has a different energy-dependent response due to the thickness of the moderator. The above-described principle was recently adopted to design and construct passive and active neutron spectrometers using only one single moderator. The unfolding processes for single sphere spectrometry systems follow standard principles described widely in the literature^(1)^.

### The passive multi-layer neutron spectrometer

**Figure 1:**
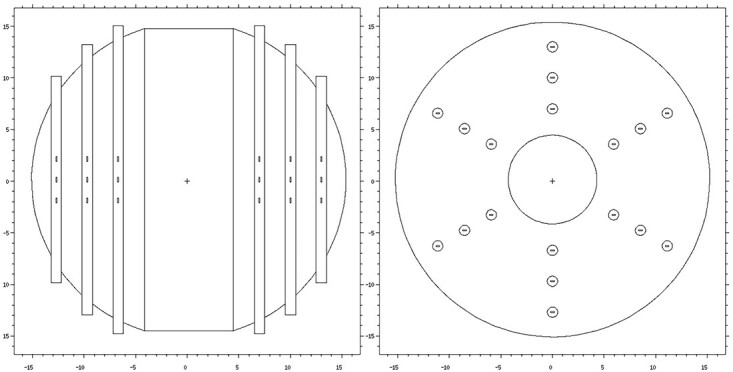
Cross-sectional views of the SWP-1 multi-layer neutron spectrometer. MCNP 6.1 plot^(4)^.

In the National Centre for Nuclear Research (NCBJ), the passive multi-layer neutron spectrometer, SWP-1 was developed^(3)^. It has a form of a 12″ high-density polyethylene sphere with six vertical slots located every 60° around the sphere at three depths corresponding to ⁓2″, 4″ and 6.5″ of moderating material (Figure 1). In the central part of the spectrometer, there is additional space for a recombination chamber for the measurements requiring monitoring of the field or extra information about the quality of the radiation. However, in the case of this work only passive measurements were performed, i.e. central slot was filled with the material equivalent to the moderator. Thermoluminescent detectors of type MTS-6 and MTS-7 were chosen for the spectrometer.

### Thermoluminescent detectors

Lithium fluoride thermoluminescence (TL) detectors developed at the Institute of Nuclear Physics of the Polish Academy of Sciences in Kraków in the form of sintered circular pellets, were chosen as passive detectors^(5)^. Two types of MTS (LiF:Mg,Ti) TL detectors were chosen i.e. MTS-6 (^6^Li enrichment above 99.9%) and MTS-7 (^7^Li enrichment to about 95%). Before the measurement, TL detectors were annealed at the temperature of 400°C for 2 h, then rapidly cooled down, annealed for 1 h at 100°C and finally cooled down to room temperature. After irradiation, detectors were read out using the MICROLAB Manual Reader-Analyzer TL RA’94^(5)^. The number of counts i.e. number of ^6^Li(n,t) reactions in the MTS-6 detectors have been corrected for gamma radiation (via MTS-7) and background radiation (non-irradiated reference TL detectors).

The aim of this work was to test the passive multi-layer neutron spectrometer in a calibration laboratory with ^239^Pu–Be and ^252^Cf isotopic sources. The paper presents steps to get the neutron spectra using unfolding methods. Additionally, Monte Carlo simulations were performed to provide response functions for TLD detectors and default spectra in the calibration laboratory.

## Materials and methods

To perform BSS-like passive neutron spectrometry (BSS stands for Bonner Sphere Spectrometer) usually one has to perform the following steps:

- Monte Carlo-based calculations of the detector’s response functions using e.g. MCNP code
- Selection or calculation of the guess spectrum at the point of measurement
- Calibration of detectors at the reference field
- Measurements at the point of interest
- Detectors readout and corrections
- Post-processing i.e. unfolding of the spectrum using dedicated tools e.g. MAXED or GRAVEL codes

### Monte Carlo simulations

To calculate the initial guess spectra of ^239^Pu–Be and ^252^Cf in calibration room and to get the response functions of TL detectors placed in the SWP-1 spectrometer the Monte Carlo N-Particle Transport Code, MCNP^(4)^ was used. Table 1 presents the simulation parameters used in this study, in the form recommended by the American Association of Physicists in Medicine, AAPM TG286^(6)^.

**Table 1:** Monte Carlo methods table as recommended by AAPM TG286.

Parameter	Description	References
Code, version	Monte Carlo N–Particle Transport Code System, MCNP6.1	^(^ ^4^ ^)^
Validation	Validation Suites for MCNP	^(^ ^8^ ^)^
Hardware	Intel(R) Core(TM) i7-8550U CPU @ 1.80GHz, 1992 MHz and Swierk Computing Centre (CIŚ) https://www.ncbj.gov.pl/en/cis-home	—
Source description	Monoenergetic, homogeneous, neutron, circular plane source, 12″ in diameter, 100 cm from the spectrometer center	—
Cross sections	ENDF70, ENDF70SAB and ENDF60 MCNP neutron data libraries based on ENDF/B-VII.0	^(^ ^9^ ^)^
Transport parameters	Defaults for mode n p	^(^ ^4^ ^)^
Scored quantities	Track length estimate of cell flux (tally f4); ^6^Li(n,t) reaction rate (fm tally multiplier)	^(^ ^4^ ^)^
# histories/statistical uncertainty	1E08/below 10% for track length estimate of cell flux	—

#### Response functions

Response functions for all TL detectors placed in the spectrometer’s sphere were calculated with MCNP code as a number of ^6^Li(n,t) reactions occurring in the specific sintered pellet volume. To get the response functions a track length estimate for fluence on the detector cells was used (tally F4). Then thanks to the tally multiplier (fm) it was possible to convert the fluence to the number of ^6^Li(n,t) events. Response functions were calculated for monoenergetic neutrons at an energy range from 1 meV to 100 MeV.

#### Initial guess spectra

For many unfolding programs (including those described in this work) the initial guess spectra are mandatory. Thus ^239^Pu–Be and ^252^Cf spectra were calculated with MCNP in reference point inside the calibration laboratory at NCBJ. Starting spectra for calculations were taken from International Atomic Energy Agency Compendium data^(7)^.

### Unfolding

Deconvolution was performed with UMG (Unfolding with MAXED and GRAVEL) package, version 3.3. It consists of several programs for the analysis of data measured with spectrometers that require the use of unfolding techniques^(10, 11)^. For both codes, the initial guess spectrum is needed and the final solution strongly depends on its quality. In addition to the final spectra it was possible to calculate the integral quantities, i.e. total neutron fluence, neutron fluence by energy groups, ambient dose equivalent rate H^*^(10), fluence-averaged energy and H^*^(10)-averaged-energy. Ambient dose equivalent rates were calculated using ICRP74/ICRU57 conversion coefficients^(12)^.

## Results

**Figure 2:**
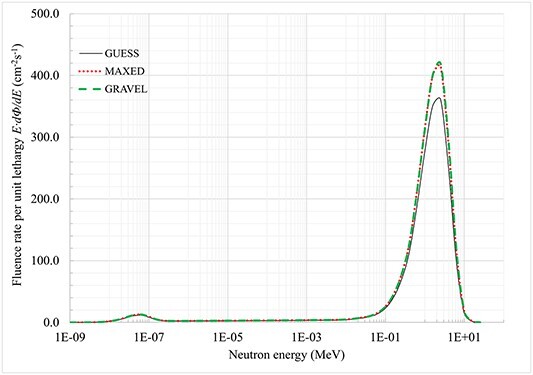
^**252**^Cf solution spectra calculated with MAXED and GRAVEL unfolding codes.

**Figure 3:**
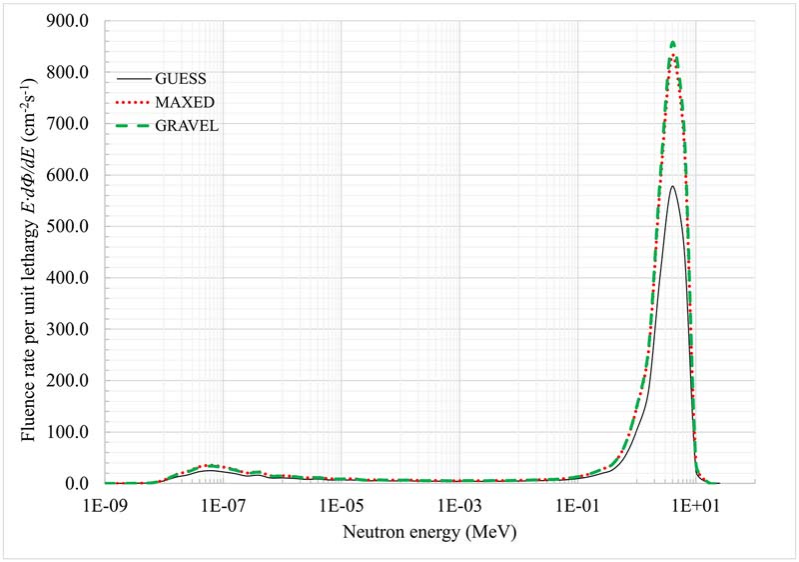
^**239**^Pu–Be solution spectra calculated with MAXED and GRAVEL unfolding codes.

^252^Cf and ^239^Pu–Be spectra calculated with MAXED and GRAVEL presented together with initial guess spectra obtained via MCNP calculations are shown in Figures 2 and 3, respectively.

In Table 2 integral quantities, i.e. total neutron fluence, neutron fluence by energy groups, ambient dose equivalent rate H^*^(10), fluence-averaged energy and H^*^(10)-averaged energy calculated on the basis of the unfolded spectra are presented. Additionally, for H^*^(10) rate and fluence rate, the reference values for measuring points were added.

**Table 2:** Integral quantities values for unfolded spectra (MXD—MAXED, GRV—GRAVEL) and reference values (REF—reference) for calibration laboratory at the reference point of measurement defined with ISO 8529^(13, 14)^.

Neutron source	^239^Pu–Be			^252^Cf		
Quantity	MXD	GRV	REF	MXD	GRV	REF
H^*^(10) rate (mSv/h)	1.829	1.875	1.440	1.309	1.316	1.214
Φ rate, total (cm^−2^ s^−1^)	1459	1483	1023	1043	1031	876
Φ rate, *E* < 0.4 eV (%)	6.31	5.93	—	2.63	2.57	—
Φ rate 0.4 eV < *E* < 0.1 MeV (%)	7.50	7.11	—	5.33	5.23	—
Φ rate, *E* > 0.1 MeV (%)	86.19	86.96	—	92.05	92.20	—
Fluence-averaged neutron energy (MeV)^(15, 16)^	3.187	3.229	3.25	1.927	1.953	2.13
H^*^(10)-averaged neutron energy (MeV)	3.735	3.750	—	2.230	2.254	—

**Figure 4:**
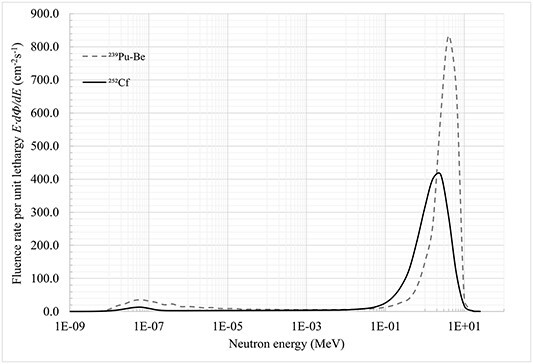
^**239**^Pu–Be and ^**252**^Cf solution spectra obtained with the MAXED unfolding code.

In Figure 4  ^239^Pu–Be and ^252^Cf unfolded spectra obtained with the MAXED unfolding code are presented together to show expected differences between the sources in the fast peak location. It should be noted that in the ^239^Pu–Be spectrum a little higher intermediate region was observed which results from higher maximum and average neutron energy of the source. The data that support the findings of this study are available from the corresponding author, [MM], upon reasonable request.

## Discussion

Since the geometry model for calculation of guess spectra with MCNP code contained a lot of laboratory room details, the qualitative information about the neutron energy spectrum seemed to be enough for unfolding. However, comparing the integral, fluence-based quantities with available reference data it was shown that guess spectra were significantly underestimated especially in the fast neutron peak region. Unfolding algorithms of MAXED and GRAVEL codes increased the fluences in the desired energy bins, but finally it resulted in overestimation of the integral fluence-based quantities (~27% and 8% in H^*^(10) rate for ^239^Pu–Be and ^252^Cf, respectively).

Taking into account the limited number of depths at which the TL detectors are located, and that uncertainties related to the TL detectors’ measurements can be significant, especially taking into account the limited number of TL detectors that can be placed in the reference point for which the response functions are calculated with Monte Carlo technique, the results are sufficient for a rough estimation of unknown field neutron spectrum and related integral quantities for radiation protection purposes.

In the unfolded spectra, the thermal peaks coming from room-scattered neutrons are visible which results from calibration laboratory conditions (sources located 1 m above the solid floor, nearest wall distance equals 2 m in two directions). The room-scattered neutron contribution in terms of H^*^(10) for ^239^Pu–Be and ^252^Cf sources in the calibration room at a distance of 1 m from neutron sources are 21 and 20%, respectively—measurements were performed with Studsvik-Alnor 2202D remmeter.

As expected, the highest intensity peak for ^239^Pu–Be is shifted to higher neutron energy reaching its maximum ⁓4 MeV with a mean energy ⁓3.2 MeV. For ^252^Cf neutrons reach the maximum intensity of ⁓2.5 MeV, and the mean energy ~1.9 MeV. For both sources, the mean energy and the highest intensity peak at the measuring point are lower than in the literature^(15, 16)^ that is caused by the in-room neutron scattering and thermal neutron contribution. It justifies the use of the shadow cone technique both in routine dosemeters calibration procedures and in experimental utilisation of the neutron sources.

## Conclusion

SWP-1 neutron spectrometer in passive-only configuration was characterised in terms of response functions with MCNP code and tested with ^239^Pu–Be and ^252^Cf isotopic sources. UMG package was used to perform the unfolding procedure with MAXED and GRAVEL codes. ^239^Pu–Be and ^252^Cf spectra were obtained and the results indicate that the device can be successfully used for a rough estimation of neutron spectrum and related integral quantities.
